# Supplementary material for: Stacking Model‐Based Classifiers for Dealing With Multiple Sets of Noisy Labels
Source: Biom J. 2025 Mar 12;67(2):e70042. doi: 10.1002/bimj.70042 (PMC11898607; doi:10.1002/bimj.70042)
Supplement: Supplementary file 1 — Supporting Information [file BIMJ-67-e70042-s001.zip › density-based-ensemble-model-main/competitors_real_data/netlab-master/netlab-master/help/hmc.htm]

Netlab Reference Manual hmc


# hmc

## Purpose

Hybrid Monte Carlo sampling.

## Synopsis

```
samples = hmc(f, x, options, gradf)
samples = hmc(f, x, options, gradf, P1, P2, ...)
[samples, energies, diagn] = hmc(f, x, options, gradf)
s = hmc('state')
hmc('state', s)
```

## Description

`samples = hmc(f, x, options, gradf)` uses a
hybrid Monte Carlo algorithm to sample from the distribution `p ~ exp(-f)`,
where `f` is the first argument to `hmc`.
The Markov chain starts at the point `x`, and the function `gradf`
is the gradient of the `energy' function `f`.

`hmc(f, x, options, gradf, p1, p2, ...)` allows
additional arguments to be passed to `f()` and `gradf()`.

`[samples, energies, diagn] = hmc(f, x, options, gradf)` also returns
a log of the energy values (i.e. negative log probabilities) for the
samples in `energies` and `diagn`, a structure containing
diagnostic information (position, momentum and
acceptance threshold) for each step of the chain in `diagn.pos`,
`diagn.mom` and
`diagn.acc` respectively. All candidate states (including rejected ones)
are stored in `diagn.pos`.

`[samples, energies, diagn] = hmc(f, x, options, gradf)` also returns the
`energies` (i.e. negative log probabilities) corresponding to the samples.
The `diagn` structure contains three fields:

`pos` the position vectors of the dynamic process.

`mom` the momentum vectors of the dynamic process.

`acc` the acceptance thresholds.

`s = hmc('state')` returns a state structure that contains the state of the
two random number generators `rand` and `randn` and the momentum of
the dynamic process. These are contained in fields
`randstate`, `randnstate`
and `mom` respectively. The momentum state is
only used for a persistent momentum update.

`hmc('state', s)` resets the state to `s`. If `s` is an integer,
then it is passed to `rand` and `randn` and the momentum variable
is randomised. If `s` is a structure returned by `hmc('state')` then
it resets the generator to exactly the same state.

The optional parameters in the `options` vector have the following
interpretations.

`options(1)` is set to 1 to display the energy values and rejection
threshold at each step of the Markov chain. If the value is 2, then the
position vectors at each step are also displayed.

`options(5)` is set to 1 if momentum persistence is used; default 0, for
complete replacement of momentum variables.

`options(7)` defines the trajectory length (i.e. the number of leap-frog
steps at each iteration). Minimum value 1.

`options(9)` is set to 1 to check the user defined gradient function.

`options(14)` is the number of samples retained from the Markov chain;
default 100.

`options(15)` is the number of samples omitted from the start of the
chain; default 0.

`options(17)` defines the momentum used when a persistent update of
(leap-frog) momentum is used. This is bounded to the interval [0, 1).

`options(18)` is the step size used in leap-frogs; default 1/trajectory
length.

## Examples

The following code fragment samples from the posterior distribution of
weights for a neural network.

```
w = mlppak(net);
[samples, energies] = hmc('neterr', w, options, 'netgrad', net, x, t);
```

## Algorithm

The algroithm follows the procedure outlined in Radford Neal's technical
report CRG-TR-93-1 from the University of Toronto. The stochastic update of
momenta samples from a zero mean unit covariance gaussian.

## See Also

`metrop`

---

**Pages:**
Index


---

Copyright (c) Ian T Nabney (1996-9)
